# Distal radial access for complex percutaneous coronary interventions: current evidence and future perspectives

**DOI:** 10.1007/s12928-025-01230-6

**Published:** 2025-12-20

**Authors:** Juan F. Iglesias, Adel Aminian, Gregor Leibundgut, Claudiu Ungureanu, Grigorios Tsigkas, Giuseppe Colletti, Maarten A. H. van Leeuwen, Ivo Bernat, Stefan Harb, Karsten Schenke, Ioannis Skalidis, Arnaud Planchat, Pierfrancesco Agostoni, Gregory A. Sgueglia

**Affiliations:** 1 Department of Cardiology, Geneva University Hospitals, Rue Gabrielle-Perret-Gentil 4, Geneva, 1205 Switzerland; 2 Department of Cardiology, Centre Hospitalier Universitaire de Charleroi, Charleroi, Belgium; 3 Department of Cardiology, Basel University Hospital, Basel, Switzerland; 4 Department of Cardiology, Jolimont Hospital, La Louvière, Belgium; 5 University of Patras, Patras, Greece; 6 Cardiovascular Department, Clinique Saint Joseph, Vivalia, Arlon, Belgium; 7 Department of Cardiology, Isala Heart Center, Zwolle, the Netherlands; 8 Department of Cardiology, Faculty of Medicine Pilsen, University Hospital, Charles University, Pilsen, Czech Republic; 9 Department of Cardiology, Medical University of Graz, Graz, Austria; 10 Department of Cardiology, Agaplesion Bethesda Krankenhaus Bergedorf, Hamburg, Germany; 11 Institut Cardiovasculaire Paris Sud, Massy, France; 12 HartCentrum, Ziekenhuis Aan de Stroom (ZAS) Middelheim, Antwerp, Belgium; 13 Division of Cardiology, Sant’Eugenio Hospital, Rome, Italy

**Keywords:** Distal radial access, Complex percutaneous coronary intervention, Chronic total occlusion, Left main, Calcified lesions, Bifurcations

## Abstract

Distal radial access (DRA) is a valid alternative to conventional transradial access (TRA) for coronary angiography and/or percutaneous coronary intervention (PCI). Increasing experience and expertise with DRA have enabled its potential use in patients undergoing PCI for more complex coronary lesions, such as chronic total occlusions, left main coronary artery disease, heavily calcified lesions, and complex bifurcation lesions. However, randomized evidence on DRA is currently limited to diagnostic procedures and/or PCI of non-complex coronary lesions, typically using ≤ 6-French (Fr) arterial sheaths or guide catheters. Data regarding the feasibility and safety of DRA for complex PCI remains restricted to small, single-center studies and multicenter registries, which have included only a limited number of patients undergoing PCI with large-bore (≥ 7-Fr) guide catheters. Despite these limitations, existing data suggest that DRA is both feasible and safe for complex PCI. However, dedicated randomized trials are needed to further substantiate these findings. The DISCO COMPLEX (*NCT05490238*) randomized trial is currently investigating the superiority of DRA over conventional TRA in terms of radial artery occlusion rates in patients undergoing complex large-bore PCI with a 7-Fr introducer sheath. This article reviews the current evidence and explores future directions for DRA in complex coronary interventions.

**Supplementary Information:**

The online version contains supplementary material available at 10.1007/s12928-025-01230-6.

## Introduction

The radial artery is the recommended vascular access for coronary angiography and/or percutaneous coronary intervention (PCI) in all patient and lesion subsets, provided there are no procedural contraindications [1–3]. Compared to transfemoral access (TFA), a transradial access (TRA) significantly reduces the risk of major bleeding, vascular complications, major adverse cardiovascular events (MACE), and all-cause mortality [4, 5]. In addition, TRA enhances patient comfort and reduces healthcare costs [6]. The increasing adoption of TRA has significantly expanded its application in patients undergoing PCI for complex coronary lesions, such as chronic total occlusions (CTO), left main coronary artery (LMCA) disease, severely calcified lesions, and complex bifurcations. Although certain interventions can be safely and efficiently performed with 5- or 6-French (Fr) guide catheters, the small diameter of the radial artery has often been regarded as a limitation during PCI for complex coronary lesions, particularly when large-bore (≥ 7-Fr) guide catheters are required to optimize procedural outcomes [7]. However, a recent randomized trial demonstrated that large-bore TRA with 7-Fr guide catheters in patients undergoing complex PCI significantly reduced clinically relevant access site bleeding and vascular complications compared to TFA, without compromising procedural success [8].

Distal radial access (DRA) has emerged as a valid alternative to conventional TRA for coronary angiography and/or PCI [9]. Compared to conventional TRA, DRA is associated with lower rates of radial artery occlusion (RAO) and clinically significant hematomas [10, 11]. However, most randomized trials comparing DRA and TRA have focused on low-risk patients undergoing diagnostic angiography or PCI of non-complex lesions with 5- or 6-Fr sheaths [12–14]. As a result, these findings may not be directly applicable to higher-risk patients undergoing PCI of complex coronary lesions, which may require large-bore guide catheters to provide adequate support or accommodate specialized devices. Additionally, the smaller caliber of the distal radial artery raises concerns regarding procedural feasibility, patient comfort, and the risk of local vascular complications, particularly when large-bore sheaths are needed. It therefore remains essential to determine whether DRA can reliably support complex PCI without compromising safety or procedural success. This review provides a comprehensive analysis of the available evidence and future perspectives on the role of DRA in complex PCI.

## Vascular access for complex coronary interventions

**Fig. 1:**
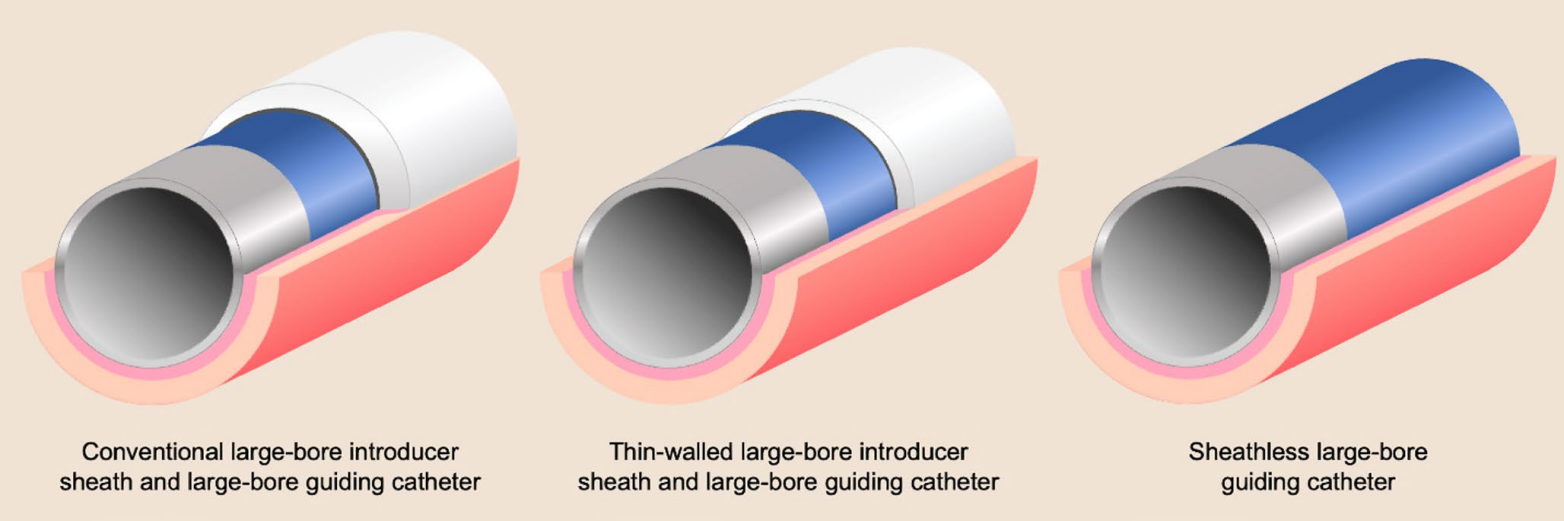
Cross-sectional schematic of a large-bore catheter in the small-diameter distal radial artery. A conventional radial sheath stretches the arterial wall, whereas the stress on the artery is minimal or absent with thin-walled introducer sheaths and sheathless approaches

The evolution of vascular access strategies in complex PCI has been driven by the dual imperative of optimizing procedural success while minimizing access site-related complications. TFA has long been the preferred vascular access site for PCI of complex coronary lesions, due to its perceived advantages in back-up support, the extensive operator experience, and the availability of guide catheters specifically designed for this approach. Moreover, the large diameter of the femoral artery, which readily accommodates large-bore guide catheters, has facilitated the use of specialized equipment for complex PCI. In contrast, the adoption of TRA in complex PCI has traditionally been constrained by the smaller caliber of the radial artery, which increases the sheath-to-artery ratio and, consequently, the risk of vascular injury, including RAO⁹. However, the advent of innovative vascular access technologies, such as thin-walled arterial sheaths and sheathless systems designed to minimize sheath-to-artery diameter mismatch, has significantly broadened the feasibility of TRA. These advancements allow standard guide catheters to be used with an outer diameter reduced by approximately one French size (Fig. 1), enabling PCI to be performed safely and effectively via a radial access across the full spectrum of coronary lesion complexity, even in patients who would traditionally require large-bore guide catheters. This *slender* PCI approach represents a paradigm shift toward minimizing vascular injury while preserving procedural efficacy and safety. Guided by the ‘*less-is-more*’ principle, this strategy prioritizes the smallest sheath and catheter size necessary (typically 5-Fr or 6-Fr guide catheters) and favors sheathless vascular systems over large-bore 7- or 8-Fr guide catheters when additional inner-lumen support is required, thereby extending the feasibility of a radial access to increasingly complex coronary interventions. While many complex procedures can be safely and efficiently performed with a 5-Fr or 6-Fr approach [15], large-bore guide catheters (≥ 7-Fr) may still be required to accommodate specialized equipment, such as calcium modification devices [15], and to optimize procedural success in selected cases [7].

Several studies have demonstrated the safety and feasibility of large-bore TRA for complex PCI. In a prospective, multicenter study including 60 patients undergoing complex PCI, TRA using the 7-Fr Glidesheath Slender introducer sheath (*Terumo Corp.*,* Tokyo*,* Japan*) was associated with a high procedural success rate and low rates of major complications, including major bleeding, major vascular complications, symptomatic radial artery spasm (RAS), and RAO at discharge [16]. The *Complex Large-Bore Radial Percutaneous Coronary Intervention* (COLOR) trial was the first prospective, randomized controlled study to compare large-bore TRA using the 7-Fr Glidesheath Slender sheath with TFA in 388 patients undergoing complex PCI [8]. At hospital discharge, large-bore TRA significantly reduced the rates of the primary composite endpoint, which included clinically relevant access site bleeding or vascular complications requiring intervention [8]. This difference was primarily driven by a lower risk of major bleeding and vascular complications, while no significant differences were observed in procedural success, access site crossover rates, procedural duration, contrast volume, or radiation exposure [8]. A recent meta-analysis demonstrated that dedicated sheathless vascular systems improve access-related efficacy compared with sheath-based strategies, while subgroup analyses suggest comparable outcomes when thin-walled arterial sheaths are used [17].

## Distal radial access for complex coronary interventions

Supported by consistent findings from multiple single-center and multicenter randomized trials, DRA has matured into a valid alternative to conventional TRA for coronary angiography and PCI [12–14]. A recent meta-analysis including 6,208 patients from 14 randomized controlled trials showed that DRA reduces the risks of RAO and large hematomas compared to TRA [10]. However, these benefits are counterbalanced by longer radial artery cannulation and sheath insertion times, more puncture attempts, and a three-fold higher risk of access site crossover [10]. Importantly, most of the trials predominantly enrolled low-risk patients undergoing diagnostic coronary angiography and/or non-complex PCI, typically using 5- or 6-Fr guide catheters. Accordingly, currently available evidence on DRA for complex PCI remains limited and is summarized in ***Supplementary Table 1***.

The *Distal vs. Conventional Radial Access* (DISCO RADIAL) trial was the first prospective, multicenter, international, randomized controlled trial to compare DRA with conventional TRA among 1,309 patients undergoing coronary angiography and/or PCI using a 6-Fr Glidesheath Slender thin-walled sheath and with strict adherence to best-practice recommendations for RAO prevention [14]. At hospital discharge, no significant difference was observed in the rate of forearm RAO [14], but DRA was associated with a shorter hemostasis time, albeit with a higher rate of access site crossover [14]. There was no significant treatment interaction between access site and the incidence of RAS or access site complications [14]. Nevertheless, the generalizability of these findings to higher-risk patients undergoing complex PCI is limited, as only a minority (36%) of enrolled patients underwent PCI, with a small proportion undergoing intervention for complex coronary lesions, such as LMCA-PCI (9%), and no use of large-bore sheaths in compliance with the trial protocol [14].

**Fig. 2:**
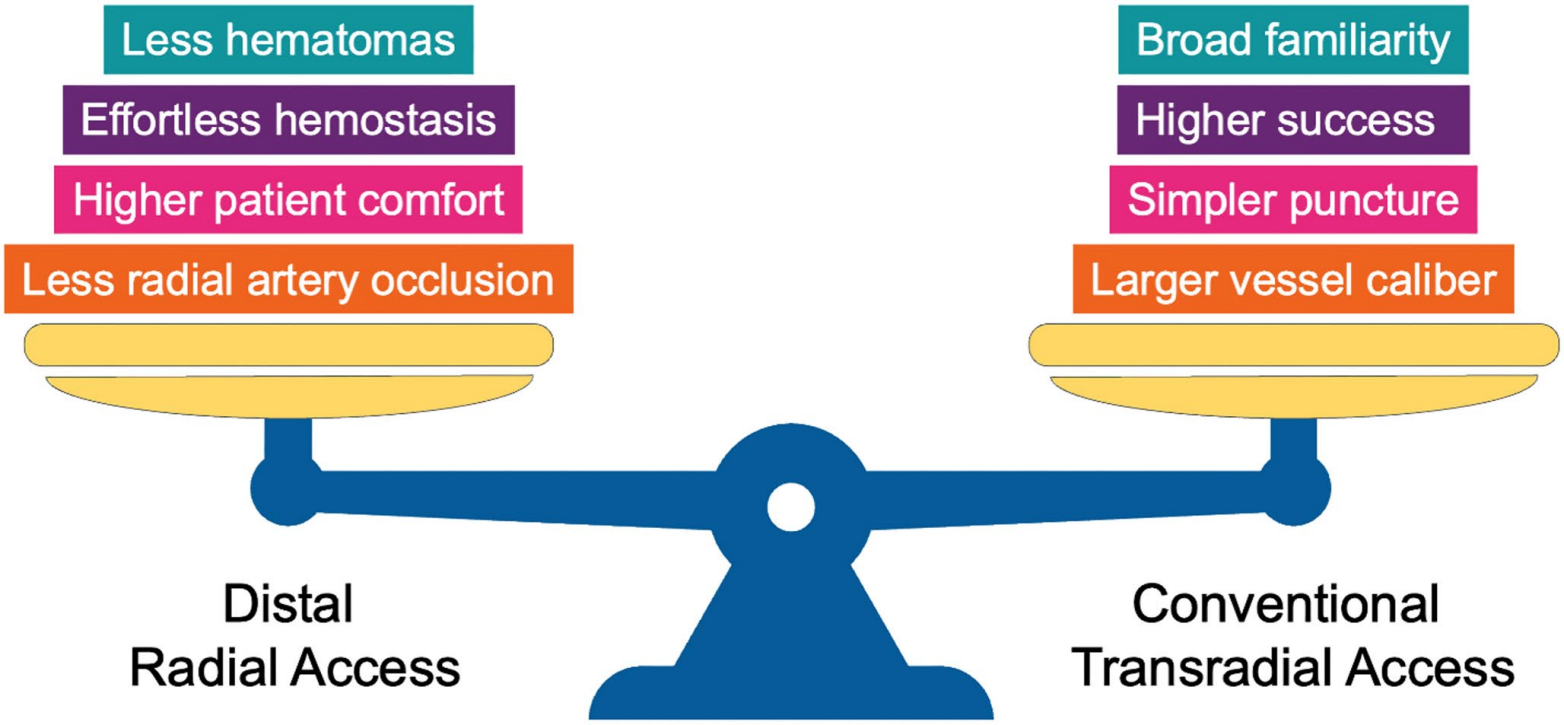
Advantages and disadvantages of distal versus conventional transradial artery access for complex percutaneous coronary intervention

The feasibility of large-bore DRA for complex PCI may be limited by the smaller caliber of the distal radial artery compared to its forearm counterpart [9] (Fig. 2). Although certain interventions have been shown to increase distal radial artery diameter, this anatomical limitation may heighten the risk of RAS, access-site crossover and RAO when large-bore (≥ 7-Fr) sheaths are used [18]. In the absence of randomized data, current evidence is primarily based on small observational studies and post-hoc analyses of observational registries. These findings require cautious interpretation due to heterogeneity in baseline patient risk, variations in the use of large-bore sheaths and guide catheters, differing study endpoints, and inconsistent methods for RAO assessment. In a prospective, single-center study including 20 patients who underwent large-bore DRA using the 7-Fr Railway sheathless vascular access system (*Cordis Corporation*,* USA*) for complex PCI (distal LMCA disease, 40%; complex non-LMCA bifurcations, 35%); CTO, 20%), of whom many patients required rotational atherectomy (RA, 45%) and intravascular lithotripsy (10%), procedural success rate was high, with no access site crossover, major bleeding, vascular complications, or distal and forearm RAO at 24 hours [19]. Severe RAS occurred in only 5% of patients [19]. A single-center, retrospective, observational study of 102 patients undergoing complex PCI (LMCA, 34%; CTO, 25%, bifurcations, 64%; RA, 11%) via DRA using the 7-Fr Braidin (*APT Medical*,* China*) thin-walled introducer sheath reported high puncture and procedural success rates [20]. The incidences of forearm RAO (2.2%), RAS (2.2%), and vascular complications (1.1%) were low [20]. In a real-world registry of 1,692 patients, PCI with ≥ 7-Fr access was performed in 36% of cases, with only 4.7% requiring crossover to a larger-diameter vessel [21]. Recently, a prospective, single-center, observational study which included 100 Korean patients treated with PCI, of whom 51% underwent complex PCI (CTO, 14%; LMCA, 13%; bifurcations, 6%), found that DRA using the 7-Fr Prelude Ideal (*Merit Medical*,* USA*) thin-walled introducer sheath was associated with high procedural success, minimal access-site complications, and no cases of proximal RAO [22]. However, isolated cases of additional vascular complications following DRA in complex PCI have been reported, including pseudoaneurysms, managed conservatively [23] or with percutaneous thrombin injection [24], and arteriovenous (AV) fistulas requiring surgical repair [25].

**Fig. 3:**
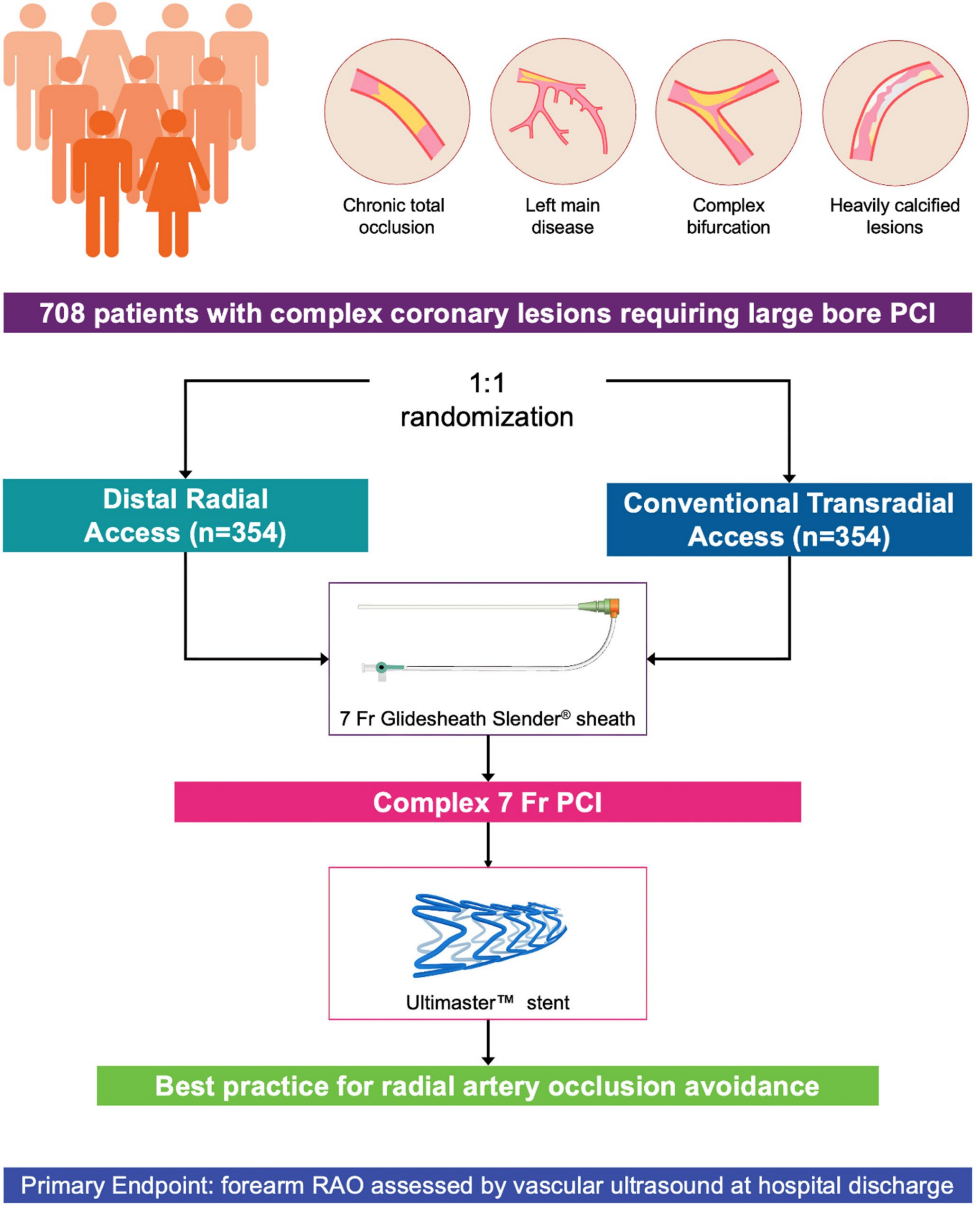
Outline of the DISCO COMPLEX randomized controlled trial. Fr, French; PCI, percutaneous coronary intervention; RAO, radial artery occlusion

These promising findings suggest that large-bore DRA, when performed by experienced operators and with dedicated vascular access systems, may be a safe and effective approach for complex PCI. However, larger randomized controlled trials are needed to confirm the safety and efficacy observed in these preliminary studies. The ongoing *DIStal Versus COnventional Radial Access for COMPLEX Large-bore Percutaneous Coronary Intervention* (DISCO COMPLEX) (*NCT05490238*) investigator-initiated, prospective, multicenter, international, open-label, randomized, controlled trial is the first study evaluating the superiority of large-bore DRA over conventional TRA using the 7-Fr Glidesheath Slender thin-walled sheath in reducing forearm RAO at hospital discharge among 708 patients undergoing complex PCI with large-bore guide catheters [26] (Fig. 3). The results of this trial are expected to provide the first definitive randomized evidence on the safety and efficacy of large-bore DRA in this high-risk population, with the potential to broaden its adoption for complex PCI.

## Distal radial access in complex coronary lesion subgroups

### Chronic total occlusions

In addition to optimal medical therapy, PCI and coronary artery bypass grafting are potential revascularization options for selected patients with CTOs, with the choice guided by a multidisciplinary heart team based on patient-specific anatomical and clinical factors. CTO-PCI has traditionally been performed via TFA to enable the use of large-bore guide catheters that improve procedural success rates [7]. However, TRA for CTO-PCI has progressively gained popularity despite initial concerns regarding its safety and efficacy, as well as the limited clinical evidence [27]. In a meta-analysis including 10,590 patients from 9 observational studies who underwent CTO-PCI, TRA demonstrated technical success and in-hospital mortality rates comparable to those of TFA but was associated with lower risks of access site complications and major bleeding [28]. Due to the non-randomized nature of the included studies, TRA was predominantly attempted in lower-risk patients with less complex CTO lesions. FORT-CTO is the first dedicated randomized trial designed to compare TRA versus TFA among 610 CTO patients, of whom 76% were treated with large-bore (7-Fr) guide catheters [29]. TRA showed similar procedural success rates, procedure duration, contrast volume use, and radiation dose exposure compared to TFA, but was associated with significantly fewer access site complications.

DRA for CTO-PCI is increasingly adopted [30], but the available evidence remains scarce, despite CTO representing the complex PCI subset with the most published data. In a large-scale, prospective, multicenter study including 4,977 Korean patients treated via DRA, of whom 1,640 (33%) underwent PCI using mainly 5- or 6-Fr guide catheters (7-Fr, 6%; sheathless 6.5-Fr, 11%), CTO-PCI was performed in only 3% of the patients [31]. Evidence for DRA in CTO-PCI comes from case reports [32] including some describing the feasibility and safety of bilateral DRA for complex CTO-PCI [33], as well as from prospective and retrospective observational studies and post-hoc subgroup analyses of large-scale registries [29, 34–37]. The interpretation of the available evidence is limited by nonrandomized study designs, small sample sizes, variability in arterial sheath types and sizes used, and heterogeneity in study endpoint definitions, particularly in terms of technical and procedural success rates. Despite methodological limitations, these findings suggest the feasibility and safety of DRA for CTO-PCI, even when large-bore guide catheters are required.

In a prospective, multicenter, observational study involving 41 patients undergoing CTO-PCI, left DRA using a 7-Fr Glidesheath Slender sheath was associated with high procedural and technical success rates, with access site crossover occurring in 17% of patients, primarily due to weak/absent pulse or vessel tortuosity [34]. Notably, no major bleeding, vascular complications, RAS, or distal RAO assessed by vascular ultrasound at 24 h were reported, whereas the rate of distal RAO at one-month follow-up was 4.3%^34^. A direct comparison of 120 patients who underwent DRA (6-Fr, 59%; 7-Fr, 39%) and 2,625 patients treated via conventional TRA for CTO-PCI demonstrated similar technical success rates, but significantly higher procedural success rates with DRA, a finding that is likely attributable to the lower CTO complexity observed in this patient group [30]. Moreover, DRA was associated with reduced radiation exposure and fewer coronary perforations, while rates of MACE, access site complications, and major bleeding were comparable between groups [30]. Unfortunately, RAO rates were not reported. In a single-center Chinese registry of 298 patients undergoing DRA for CTO-PCI primarily using a Glidesheath Slender sheath (7-Fr, 19%), technical and procedural success rates were high, with access site complications reported in < 1% of patients [35]. The reported RAO rate was extremely low (0.5%), but the assessment method was not specified. In a retrospective, multicenter study, DRA for CTO-PCI was associated with lower contrast volume and reduced radiation exposure, but with longer procedural and fluoroscopy times compared to conventional TRA [36]. Procedural and clinical success rates were high and comparable between access strategies, with numerically fewer vascular complications including RAO (1.3% vs. 2.7%) in the DRA group, despite a greater CTO complexity [36].

### Left main coronary artery disease

LMCA-PCI presents several technical challenges due to the larger diameter of the vessel and the frequent involvement of distal bifurcation lesions, which may require large-bore guide catheters to accommodate one or more large-size balloons. TFA has traditionally been preferred for LMCA-PCI due to the perceived limitations of TRA, including limited compatibility with large-bore devices and restricted feasibility for hemodynamic support when needed. In a meta-analysis of 12 non-randomized studies including 17,258 patients (TRA, 46%) undergoing LMCA-PCI, TRA significantly reduced major bleeding, access site bleeding, access site and vascular complications, and in-hospital mortality, without compromising procedural success [38]. Clinical evidence on large-bore TRA for LMCA-PCI remains limited. In a retrospective, single-center Chinese study of 82 patients with LMCA bifurcation lesions treated via TRA using a 7.5-Fr sheathless guide catheter, procedural success was high, while vascular complications were low [39].

There is currently no dedicated randomized data on the safety and efficacy of DRA for LMCA-PCI. Furthermore, the limited representation of LMCA-PCI cases performed via DRA in randomized trials and large-scale registries restricts the generalizability of current findings to this higher-risk subgroup. In the DISCO RADIAL trial, LMCA-PCI via DRA using a 6-Fr sheath was performed in only 9% of patients [14]. Similarly, in a large-scale multicenter Korean registry, LMCA-PCI via DRA was performed in only 3% of patients with limited use of large-bore guide catheters in the overall cohort [30]. Available evidence is primarily derived from reports describing successful procedures using 6-Fr [40–42] or 7-Fr [43] guide catheters for complex LMCA-PCI.

### Non-left main complex bifurcation lesions

TRA represents a viable alternative to TFA for non-LMCA bifurcation PCI. Provisional side branch stenting remains the preferred strategy and can typically be performed using 5- or 6-Fr guide catheters, which are compatible with most radial artery sizes. In a Korean registry including 1,668 patients who underwent non-LMCA bifurcation PCI (true bifurcations, 70%; provisional stenting, 83%), there were no significant differences between TRA (using mainly 6-Fr guide catheters) and TFA in procedural success rates and clinical outcomes [44]. More complex bifurcations requiring two-stent PCI techniques [1–3] or large balloons for kissing-balloon dilatation may be less practical with 5- or 6-Fr guide catheters due to equipment size constraints. In a retrospective study of 805 patients with true bifurcations undergoing PCI with a two-stent approach, TRA was associated with similar in-hospital outcomes, shorter hospitalization, reduced bleeding, and comparable long-term clinical safety and efficacy compared to TFA [45]. However, data on large-bore 7-Fr TRA for complex non-LMCA bifurcation PCI is scarce [8, 16].

Evidence on the feasibility and safety of DRA for complex non-LMCA bifurcation PCI remains limited. In a real-world registry of 1,000 DRA procedures performed without large-bore sheaths, complex bifurcations accounted for 17% of the 356 PCIs performed [46]. A single-center observational study including 106 patients undergoing bifurcation PCI via left DRA with mostly 6-Fr guide catheters reported 100% procedural success rate, with no access site crossover, major bleeding, RAO, hematoma, or mortality at 30 days [47]. Data on large-bore (≥ 7-Fr) DRA is similarly scarce [19].

### Heavily calcified coronary lesions

TFA has traditionally been preferred for PCI of severely calcified coronary lesions because large-bore guide catheters are often required to accommodate the large RA burrs used for plaque modification. However, recent advances in RA techniques have favored smaller burr sizes, allowing RA to be performed via TRA using large-bore 7-Fr guide catheters [16] or 7.5-Fr sheathless systems [48] that are compatible with most radial artery diameters. In a meta-analysis of observational studies comprising 9,153 patients undergoing RA, TRA was associated with a 55% reduction in major access site bleeding and lower radiation exposure compared with TFA [49]. No significant differences were observed in procedural success, procedure time, length of hospital stay, in-hospital all-cause mortality, MACE, myocardial infarction, or stent thrombosis [49]. Nevertheless, reported experience with large-bore TRA for PCI of calcified lesions remains limited.

Because most equipment needed is compatible with 5-Fr (lithotripsy balloons), 6-Fr (1.25–1.5 mm RA burrs, 1.25 mm orbital atherectomy crown), and 7-Fr standard or 7.5-Fr sheathless (1.75 mm RA burr) guide catheters, DRA has emerged as a viable option for PCI of severely calcified lesions. However, evidence on its feasibility and safety remains limited [19, 50]. In a recent study comparing DRA with conventional TRA in 700 patients undergoing PCI, a 7-Fr sheath was used in 7.1% of DRA cases, whereas calcified lesions were treated in only 5.7% of DRA patients [50]. More recently, the safety and feasibility of intracoronary lithotripsy via DRA using a slender 5-Fr sheathless guide catheter approach have also been reported [51].

## Discussion

**Fig. 4:**
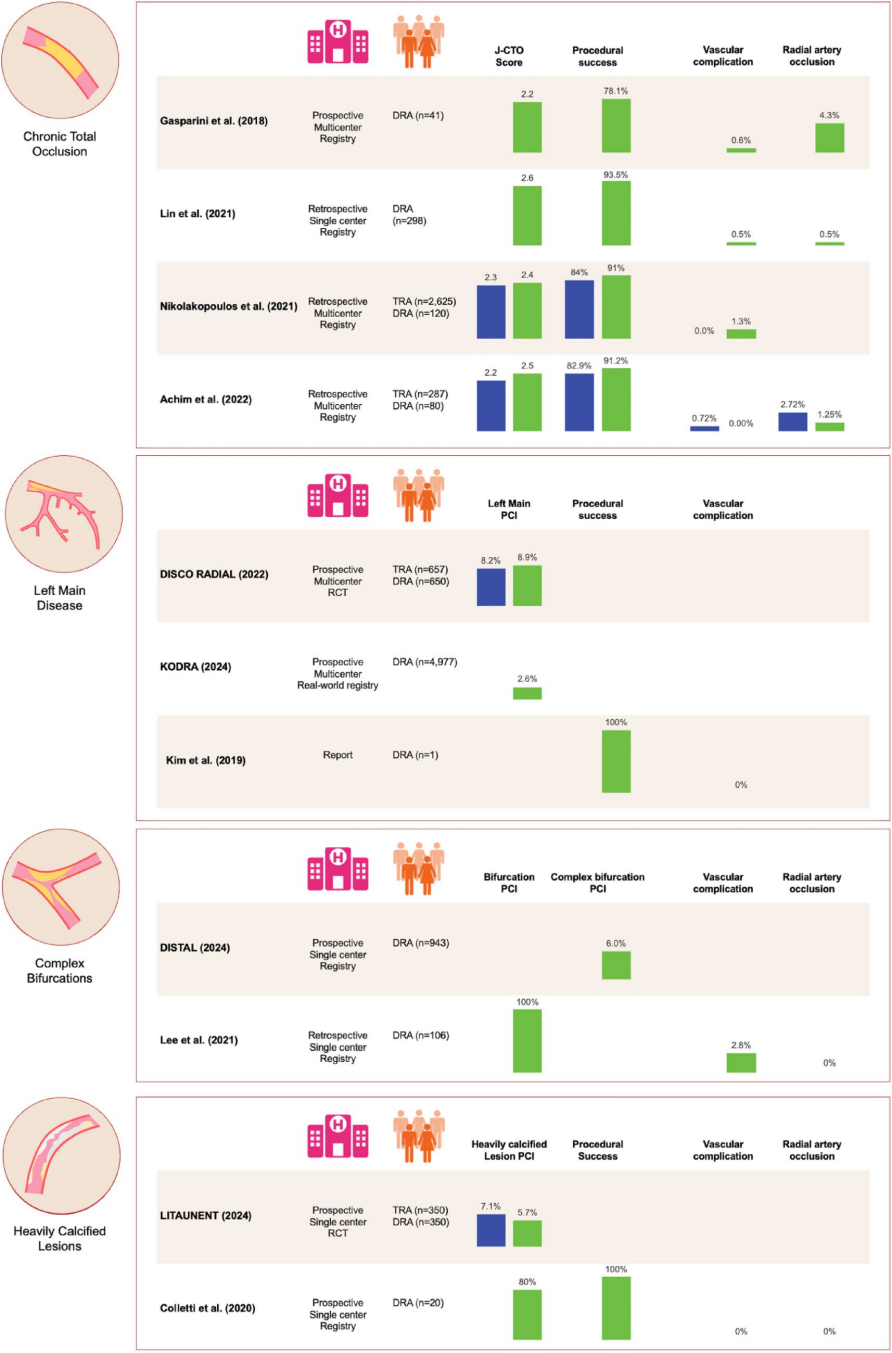
Integrated summary of available evidence on distal radial access for complex percutaneous coronary intervention. Histogram scales are not uniform and are adjusted to provide a meaningful data visualization. DRA, distal radial access; Fr, French; J-CTO, Japanese-Chronic Total Occlusion; PCI, percutaneous coronary intervention; RCT, randomized controlled trial; TRA, conventional transradial access

Optimal vascular access strategies in complex PCI are shaped by the dual imperative of maximizing procedural success while minimizing access site–related complications. Within this context, DRA has emerged as a safer and similarly effective alternative to conventional TRA and TFA, offering comparable procedural success with a lower incidence of major bleeding and vascular complications [10–14]. Nonetheless, despite growing interest and expanding adoption, contemporary evidence supporting DRA in complex coronary interventions remains limited to low-quality studies, with only a minority of patients treated using large-bore (≥ 7-Fr) guide catheters (Fig. 4).

Available evidence suggests that DRA is a promising alternative to conventional TRA in CTO-PCI, whether used bilaterally to preserve vascular access and reduce bleeding or incorporated into hybrid strategies with femoral or conventional TRA to facilitate complex large-bore techniques while minimizing vascular trauma. However, randomized comparative data remain scarce, particularly in cases requiring large-bore guide catheters. A key anatomical consideration in CTO-PCI is the distance between the access site and the coronary ostium. In specific scenarios, such as retrograde CTO interventions using epicardial collaterals or bypass grafts, shorter (90-cm) guide catheters are often necessary. In such cases, the additional length imposed by DRA may be restrictive, especially with right-sided access. In the absence of dedicated guide catheters, operators should acknowledge this limitation when selecting DRA for CTO-PCI, particularly in taller patients or those with anticipated brachiocephalic trunk tortuosity, such as older individuals or patients with arterial hypertension. Available evidence also suggests that large-bore DRA is feasible for LMCA-PCI; however, the number of treated patients, particularly those requiring ≥ 7-Fr guide catheters, remains limited, underscoring the need for dedicated comparative studies with TRA. Data supporting large-bore DRA for complex bifurcation PCI are similarly encouraging but remain preliminary. Finally, DRA appears technically feasible for the treatment of heavily calcified lesions; however, its role remains insufficiently defined, particularly when large-bore guide catheters are required to accommodate advanced calcium-modification devices. In summary, dedicated randomized trials evaluating DRA in complex PCI are urgently needed, particularly for procedures requiring large-bore guide catheters. The ongoing DISCO COMPLEX trial is designed to address this gap in knowledge by assessing whether DRA provides superior radial artery preservation compared with conventional TRA in complex large-bore PCI²⁶, potentially informing future recommendations on optimal access strategies for complex PCI.

**Fig. 5:**
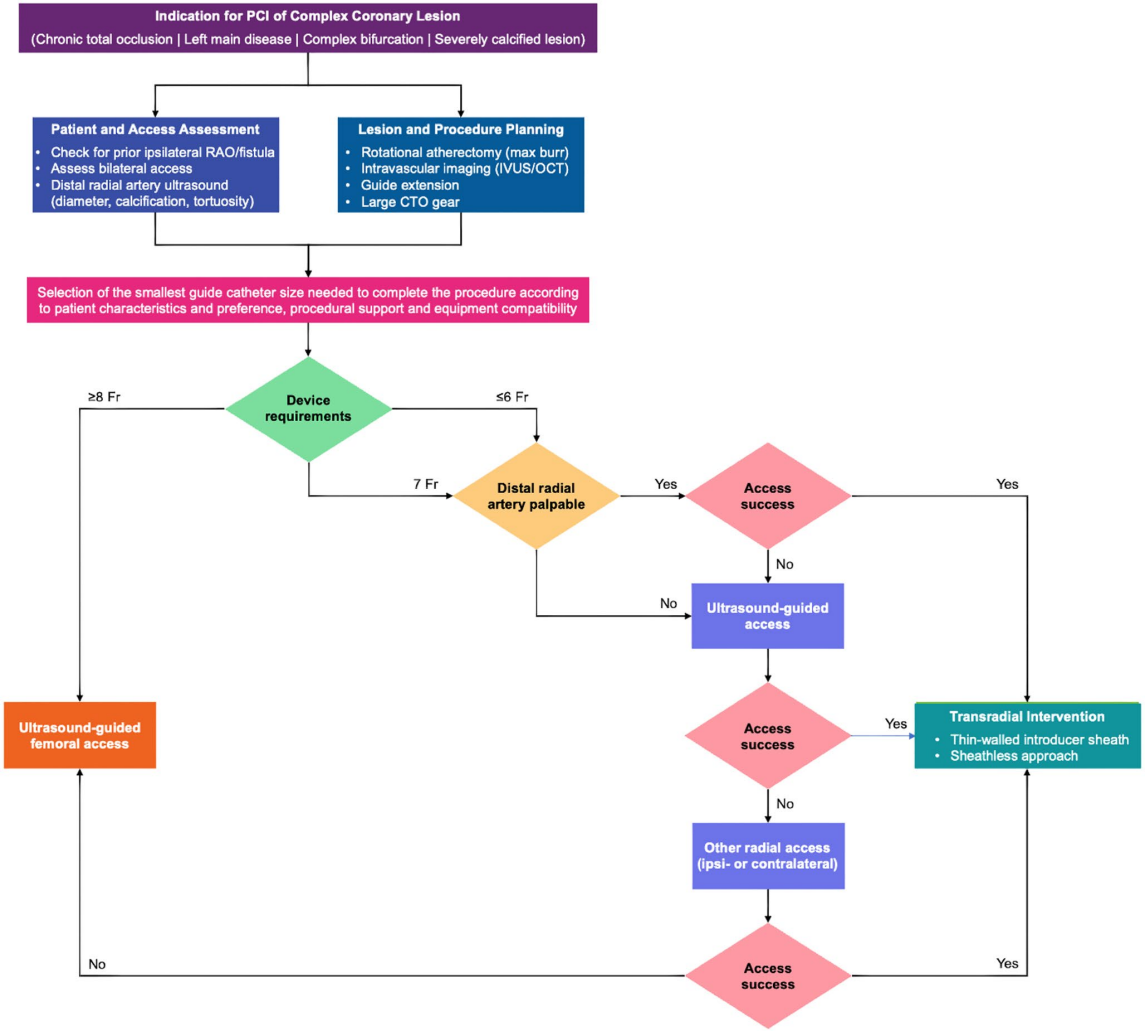
Stepwise approach to vascular access site selection in complex percutaneous coronary intervention. CTO, chronic total occlusion; Fr, French; IVUS, intravascular ultrasound; OCT, optical coherence tomography; PCI, percutaneous coronary intervention; RAO, radial artery occlusion

**Fig. 6:**
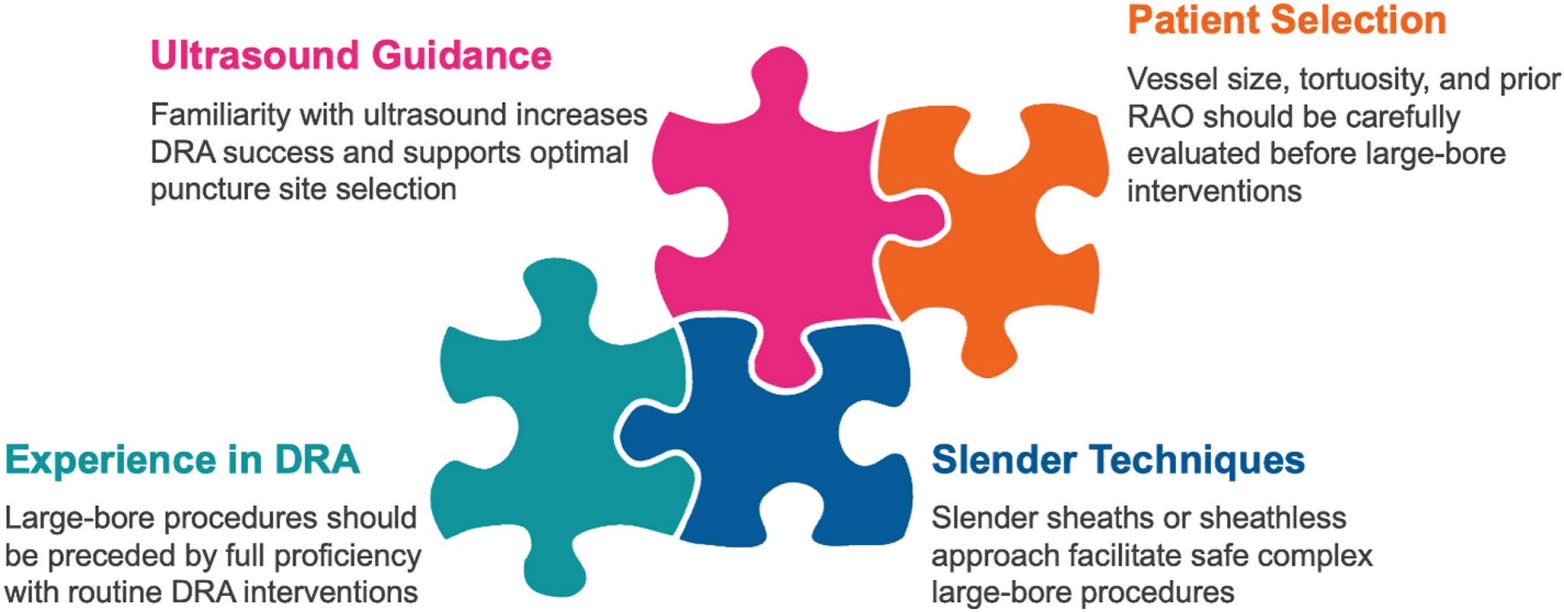
Practical recommendations for performing complex percutaneous coronary intervention via distal radial access. DRA, distal radial access; RAO, radial artery occlusion

The transition to DRA for complex PCI in clinical practice should be gradual and guided by operator experience, given the associated learning curve. Training in DRA puncture techniques, the use of ultrasound guidance, and management of access site–related complications are critical for procedural safety. A stepwise approach to vascular access site selection in complex PCI is provided in Fig. 5. Patient selection should consider prior vascular history (RAO, AV fistula), need for bilateral arterial access, distal radial artery characteristics (diameter, calcification, and tortuosity), lesion complexity, procedural requirements, and patient preference. Preprocedural ultrasound can further facilitate vascular access planning and reduce failed puncture attempts, particularly during early operator experience. A *slender* PCI strategy (selecting the smallest guide catheter size necessary to complete the procedure and utilizing dedicated thin-walled or sheathless vascular systems) should be prioritized. Practical guidance for operators performing complex PCI via DRA, including operator experience, patient selection, vascular ultrasound guidance, and slender techniques, is summarized in Fig. 6. In particular, sheathless guide catheter systems may play an important role in extending the feasibility of complex large-bore PCI via DRA [17]. By eliminating the arterial sheath, this approach maximizes usable internal diameter and may expand the scope of advanced or complex strategies. Concurrently, the development of less invasive techniques and novel low-profile coronary devices, such as 5-Fr compatible lithotripsy balloons for the treatment of calcified lesions [51], offers additional avenues to simplify complex PCI without systematically requiring large-bore guide catheters. By preserving the radial artery for future procedures, these approaches support adherence to current guideline recommendations while maximizing procedural safety.

## Limitations and future perspectives

Despite encouraging early data supporting the use of DRA for complex PCI, current evidence remains constrained by several limitations. First, the small caliber of the distal radial artery raises concerns about potential anatomical limitations of DRA across broader patient populations. Although a post-hoc subgroup analysis from the DISCO RADIAL randomized trial demonstrated consistent advantages of DRA over conventional TRA across diverse patient populations, irrespective of body weight, height, surface area, or mass index [52], these findings are derived from a predominantly low-risk cohort, with only a minority undergoing non-complex PCI using 6-Fr sheaths. As such, extrapolation to higher-risk patients undergoing large-bore complex PCI remains uncertain. Data are likewise sparse in other high-risk subgroups, including elderly patients, women with smaller vessel diameters, and individuals with diabetes mellitus, chronic kidney disease, or peripheral artery disease. The feasibility and safety of DRA for complex PCI in these populations, particularly when large-bore guide catheters are required, have yet to be clearly established.

Second, although DRA reduces the risk of RAO at short-term follow-up compared with conventional TRA [10, 11], its long-term impact on radial artery patency remains uncertain. Long-term patency may be clinically more relevant, as it determines the suitability of the radial artery for repeat vascular access or for use as a conduit in CABG or AV fistula creation. Studies of conventional TRA suggest that RAO decreases over time due to spontaneous radial artery recanalization [53]. In a randomized study comparing DRA with conventional TRA using 6-Fr sheaths, the incidence of RAO decreased from 2.5% at 24 h to 0.8% at 3 months, with long-term RAO significantly lower with DRA than with TRA [54]. Notably, two-thirds of RAO cases at 24 h in the DRA group (compared with 50% in the TRA group) recanalized spontaneously by 3 months [54]. While these findings support a more favorable long-term profile with DRA, evidence on the impact of larger-bore sheaths remains limited [55]. Further research is also needed to assess long-term patency after DRA using large-bore (≥ 7-Fr) sheaths.

Third, TRA causes acute vascular injury and long-term endothelial dysfunction in a substantial proportion of patients, leading to arterial remodeling that increases the risk of RAO and reduces the likelihood of successful repeat radial access [53–56]. Whether DRA mitigates these long-term adverse effects, particularly in the context of large-bore guide catheter use, remains to be confirmed. Similarly, although radial artery graft patency is significantly lower when the same artery has been used for angiography prior to CABG [57], the long-term advantages of DRA in this setting have yet to be established.

Fourth, although DRA is safer than conventional TRA with respect to RAO and major bleeding or vascular complications [10, 11] owing to its more favorable anatomical characteristics, evidence on less common complications, such as radial artery dissection, perforation, AV fistula, or pseudoaneurysm, remains limited, but may become clinically relevant when large-bore equipment is required.

Fifth, existing data suggest that DRA does not impair hand function when predominantly 5-Fr and 6-Fr sheaths are used [58], but experience with large-bore (≥ 7-Fr) sheaths is scarce. The DISCOPHILE COMPLEX study will provide important insight into whether DRA preserves both short- and long-term hand function in the context of complex PCI using 7-Fr sheaths and guide catheters [26]. Finally, future studies should clarify the role of ultrasound guidance in DRA to improve puncture success rates, reduce access-site crossovers, and minimize vascular complications.

## Conclusion

DRA has matured into a viable alternative to conventional TRA and TFA for complex PCI, but high-quality comparative data remain limited, particularly in patients requiring large-bore access. Although current evidence supports the feasibility and safety of DRA for complex PCI, dedicated randomized trials including patients with complex coronary lesions treated with large-bore guide catheters are still needed. The ongoing DISCO COMPLEX randomized trial aims to address this knowledge gap and may establish DRA as the preferred access site for large-bore complex PCI.

## Supplementary Information

Below is the link to the electronic supplementary material.

Supplementary Material 1

## Abbreviations

BARC: Bleeding Academic Research Consortium
CTO: Chronic total occlusion
DRA: Distal radial access
Fr: French
LMCA: Left main coronary artery
MACE: Major adverse cardiovascular events
PCI: Percutaneous coronary intervention
RA: Rotational atherectomy
RAO: Radial artery occlusion
RAS: Radial artery spasm
TFA: Transfemoral access
TRA: Transradial access
