# Supplementary material for: Distal radial access for complex percutaneous coronary interventions: current evidence and future perspectives
Source: Cardiovasc Interv Ther. 2025 Dec 20;41(2):321–33. doi: 10.1007/s12928-025-01230-6 (PMC13002766; doi:10.1007/s12928-025-01230-6)
Supplement: Supplementary file 1 — Supplementary Material 1 [file 12928_2025_1230_MOESM1_ESM.docx]

**SUPPLEMENTARY MATERIAL**

Supplementary material to: Iglesias JF, et al. *Distal radial access for complex percutaneous coronary interventions: current evidence and future perspectives.*

**TABLE OF CONTENTS**

[1. SUPPLEMENTARY TABLES 3](#_Toc207351962)

[1.1 SUPPLEMENTARY TABLE 1. Summary of main studies investigating distal radial access for complex percutaneous coronary interventions. 3](#_Toc207351963)

1. SUPPLEMENTARY TABLES

## SUPPLEMENTARY TABLE 1. Summary of main studies investigating distal radial access for complex percutaneous coronary interventions.

| **Study** | **Year** | **Design** | **Patients**  **(n)** | **Comparator arm** | **Complex coronary lesions** | **Introducer sheath used** | **Access site crossover** | **Procedural success** | **Access site major bleeding** | **Major vascular complications** | **Radial artery spasm** | **Proximal radial artery occlusion** |
| --- | --- | --- | --- | --- | --- | --- | --- | --- | --- | --- | --- | --- |
| Gasparini GL,  et al.^33^ | 2019 | Prospective, multicenter, observational | 41 | NA | CTO, 100% | 7-Fr Glidesheath Slender (*Terumo Corp, Japan*) | 17.1% | 78.1% | 0% | 0% | 0% | Distal RAO 0% (at 24h), and 4.3% (at 30 days) |
| Colletti G,  et al.^19^ | 2020 | Prospective, multicenter, observational | 20 | NA | LMCA, 40%; CTO, 20%; bifurcations, 35%; RA, 45%; IVL, 10% | 7-Fr Railway Sheathless System (*Cordis, USA*) | 0% | 95% | 0% | 0% | 5% | 0%  (at 24h) |
| Nikolakopoulos I,  et al.^29^ | 2021 | Retrospective, multicenter, observational | 120 | TRA | CTO, 100% | 6-Fr, 59%; 7-Fr 39% | NR | 91% | 0.8% | 1.3% | NR | NR |
| Lin CJ,  et. al.^34^ | 2021 | Retrospective, single-centre, observational | 298 | NA | CTO, 100% | Glidesheath Slender (*Terumo Corp, Japan*) (6-Fr, 81%; 7-Fr, 19%) | 3.1% | 94% | 0.2% | 0.2% | 0% | 0.5% |
| Lee OH,  et al.^45^ | 2021 | Retrospective, single-centre, observational | 106 | NA | Bifurcations, 100% (LMCA, 10% ; true bifurcations, 40%) | Prelude Radial (*Merit Medical, USA)* or Radiofocus Introducer II *(Terumo Corp., Japan)* (5-Fr, 3%; 6-Fr, 95%; 7-Fr, 2%) | 0% | 100% | 0% | 0% | 0% | 0% |
| Zong B,  et al.^20^ | 2022 | Retrospective, single-centre, observational | 102 | NA | LMCA, 34%; CTO, 25%; bifurcations, 64%; RA, 11% | 7-Fr thin-walled Braidin (*APT Medical, China*) | 10% | 98% | NR | 1.1% | 2.2% | 2.2%  (at 24h and 30 days) |
| Achim A,  et al.^35^ | 2022 | Retrospective, multicenter, observational | 80 | TRA | CTO, 100% | NR | NR | 91% | 0% | 0% | NR | 1.3% |
| Roh JW, et al. (SEVEN-BOX)^22^ | 2025 | Prospective, single-centre, observational | 100 | NA | Complex PCI, 51%; (CTO, 14%; LMCA, 13%; bifurcations, 6%) | 7-Fr Prelude Ideal (*Merit Medical, USA*) | 0% | 100% | 0% | 0% | NR | 0%  (at 30 days) |

CTO, chronic total occlusion; IVL, intravascular lithotripsy; LMCA, left main coronary artery; NA, not applicable; NR, not reported; PCI, percutaneous coronary intervention; TRA, transradial access; RA, rotational atherectomy; RAO, radial artery occlusion.
